# RBM10 Regulates Tumor Apoptosis, Proliferation, and Metastasis

**DOI:** 10.3389/fonc.2021.603932

**Published:** 2021-02-24

**Authors:** Yingshu Cao, Xin Di, Qinghua Zhang, Ranwei Li, Ke Wang

**Affiliations:** ^1^ Department of Respiratory Medicine, The Second Hospital of Jilin University, Changchun, China; ^2^ Department of Urinary Surgery, The Second Hospital of Jilin University, Changchun, China

**Keywords:** RNA-binding motif protein 10 (RBM10), alternative splicing, p53, numb, apoptosis, proliferation, RNA-binding motif protein 6 (RBM6), RNA-binding motif protein 5 (RBM5)

## Abstract

The RNA-binding motif protein 10 (RBM10) is involved in alternative splicing and modifies mRNA post-transcriptionally. RBM10 is abnormally expressed in the lung, breast, and colorectal cancer, female genital tumors, osteosarcoma, and other malignant tumors. It can inhibit proliferation, promote apoptosis, and inhibit invasion and metastasis. RBM10 has long been considered a tumor suppressor because it promotes apoptosis through the regulation of the MDM2-p53 negative feedback loop, Bcl-2, Bax, and other apoptotic proteins and inhibits proliferation through the Notch signaling and rap1a/Akt/CREB pathways. However, it has been recently demonstrated that RBM10 can also promote cancer. Given these different views, it is necessary to summarize the research progress of RBM10 in various fields to reasonably analyze the underlying molecular mechanisms, and provide new ideas and directions for the clinical research of RBM10 in various cancer types. In this review, we provide a new perspective on the reasons for these opposing effects on cancer biology, molecular mechanisms, research progress, and clinical value of RBM10.

## Introduction

The occurrence of cancer is the result of the imbalance of intracellular homeostasis and its multiple regulatory mechanisms. RNA alternative splicing is the key step in the regulation of gene expression after transcription. The defects of alternative splicing often appear in human tumors. Alternative splicing regulators include tumor promotors and tumor suppressors, which participate in the occurrence and development of landmark tumors by regulating different RNA subtypes (1). Therefore, alternative splicing is regarded as a new potential target for cancer treatment. RNA splicing is a highly regulated process, and the regulatory factors aiming at the splicing are strictly regulated. Among the regulatory factors, RNA binding proteins (RBPs) are the most important member. The differential transcription and post transcriptional regulation of RBP could regulate their level and activity in tumor cells. RNA-binding motif protein 10 (RBM10), one of the most important members of the RBP family, is an alternative RNA splicing factor that participates in the regulation of gene expression. Recent studies identified RBM10 as a potential candidate for the development of new therapeutics. For example, a mutation in exon 10 frequently occurs in lung adenocarcinoma and is an independent prognostic factor (2). Since most RBM10 mutations are nonsense, frameshift, or splice point mutations, most of these mutations lead to a significant decrease in *RBM10* mRNA expression (3). Previous studies identified low *RBM10* expression in many tumors, including lung, pancreatic, and breast cancer (4–6). Furthermore, RBM10 was recently reported to be a tumor suppressor that can promote tumor cell apoptosis and inhibit colonization (2). In this paper, we also discussed what is known about the mechanisms underlying the pleiotropic role of RBM10 and what still requires more in-depth research. We propose a hypothesis from the perspective of differences in RBM10 expression and molecular structure, which provide new ideas for future experimental research regarding the exact conditions in which RBM10 play a pivotal cancer-inhibiting role. In addition, in recent years, studies conducted on RBM10 have led to great changes in our understanding of RBM10 and the views of predecessors. Previous RBM10 research mainly focused on apoptosis and proliferation pathway; however, more and more studies show that it also plays an important role in metastasis and invasion. It is necessary to know the research status of RBM10 in various fields to determine whether RBM10 plays a positive or negative role in different cancers, and what possible pathways are involved. In this review, we summarize the role of RBM10 in majority of tumor tissues and cells studied so far, and we look forward to its future clinical value in the design of new-targeted drugs that regulate upstream and downstream pathways and would improve prognosis prediction.

## RNA-Binding Motif 10

RBM10, also known as s1-1, is a member of the RNA-binding protein (RBP) family and located at p11.23 on the X chromosome. Of note, in 1995, RBM10 cDNA was cloned from human bone marrow. The transcriptional length of *RBM10* is approximately 3.5 kb, which is divided into 24 exons. There are two variants of the *RBM10* gene, *RBM10v1*, and *RBM10v2*, which are translated into proteins that are 930 and 853 amino acids in length, respectively (7). Although one allele in each somatic cell is silenced during X chromosome inactivation, the remaining active allele is widely expressed in human cell lines and human tissues (8). The *RBM10* protein binds RNA, suggesting it may also have a binding affinity for single-stranded DNA. There are many potential phosphorylation sites in the RBM10 protein that affect its cellular localization and function (9).

There are many conserved RBM members of the RBP family with high sequence homology at the amino acid level, such as RBM3, RBM4, RBM5, RBM6, and RBM10. RBM10 has high sequence homology with RBM5 at the amino acid level, and both play an important role in the regulation of apoptosis (10). RBM5, RBM6, and RBM10 proteins all have two zinc finger (ZFN) structures, two RNA recognition motifs, while RRM1 and RRM2 share an octamer repeat (OCRE) region, a glycine G-patch, and three nuclear localization sequence (NLSs). RBM10 is located in the nucleus; however, proteins with molecular weights less than 60 kDa can enter and exit the nucleus freely, while high molecular weight proteins such as RBM10 must pass through the NLS to enter and exit the nucleus. The classical NLS is composed of a short, positively charged amino acid that is mainly composed of lysine or arginine residues. RBM10 contains multiple interdependent NLSs, including a classical binary NLS, an NLS located in the RRM region, and one in the OCRE region (11). In eukaryotic cells, due to the existence of a nuclear membrane, a mechanism that can mediate macromolecules to enter or leave the nucleus is essential. When proteins are transported to or from the nucleus, they are bound by transport receptors, which recognize NLS, and then the receptor-cargo complex is transported through the nuclear membrane. After the transport, the complex is separated to transport the cargo to the appropriate compartment (12). Each RBM contains two RNP regions, which are rich in serine and arginine and can be linked by any other amino acids. From the three-dimensional structure of RBPs, two RRMs form RNA-binding regions with an average size of 90 amino acids. RBPs must bind to RNA targets through molecular chemical interactions between the protein residues and RNA nucleotides (13).

Each RRM has preferred sequences. The preference order of RBM10 is poly G = poly U > poly C > poly A. The preference order of RBM5 for RNA is poly G > poly C > poly A> poly U (14). These preferences show that RBM proteins have different target genes and play different roles in alternative splicing. RRM1–ZNF coupling extends the specificity and affinity of RNA sequence recognition. RRM1 and ZNF domains are separated by forty amino acids that appear to work together to recognize UGUGUGGA or CUGUGGA sequences (15). The second RRM domain (RRM2) recognizes a C-rich sequence that can interact with intron 3 of the *Numb* exon 9, which helps to regulate the Notch pathway in tumors (16). Although the RRM2 domain of RBM5 can bind to a variety of RNA sequences with similar affinity, the sequence specificity of RBM10 for RRM2 is unclear. RRM2 is separated from the ZNF domain by a long linker, which is likely to be structurally decoupled; therefore, RRM2 can be regarded as a structure-independent unit (15).

**Figure 1:**
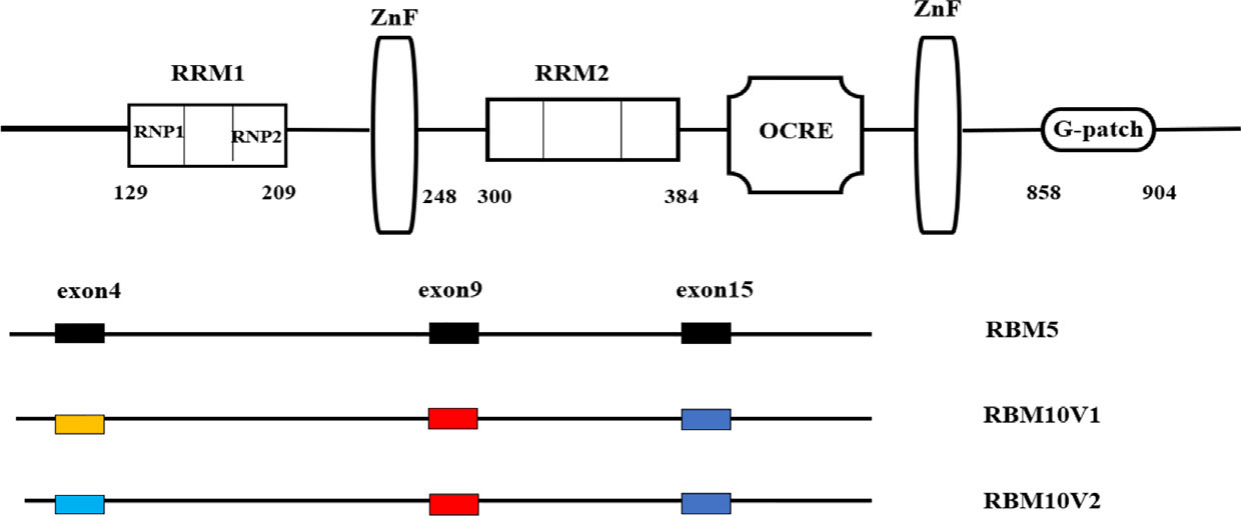
Molecular structure of the RBM10 protein. Structural differences between RBM5 and RBM10.

As an alternative splicing protein, RBM10 can be spliced at exon 4 to produce two different variants, RBM10v1 and RBM10v2, but predominantly produces the latter (17). The difference between the two variants is the valine residues in the second RRM domain. The presence or absence of the valine residues affects the *α*-helix structure of the RRM domain and thus, might affect RBM10 binding and help to explain why RBM10 has opposing effects on tumors. Some studies suggest that RBM10v1 can promote cell proliferation, while RBM10v2 can promote cell apoptosis. RBM10v1 and RBM10v2 share 60–64% homology with RBM5 when exons 4, 9 and 15 are excluded, indicating that the remaining amino acid sequences are highly conserved among the three proteins. Exons 9 and 15 are shared by the two RBM10 variants, but are different from those of RBM5, suggesting these sequences may influence the different functions of RBM10 and RBM5 (**Figure 1**) (17).

## Alternative Splicing

More than 90% of human genes, including tumor-related genes, are regulated by alternative splicing (18). Alternative splicing plays a vital role in both physiological and pathological conditions; it can produce different protein subtypes and some non-coding RNAs with different functions. Some of these variants are substrates for nonsense-mediated mRNA decay (NMD) (19). Abnormal alternative splicing can allow tumor cells to grow and develop drug resistance (20). Therefore, the understanding of RNA alternative splicing in tumor cells may enable the identification of biomarkers for diagnosis or prognosis (21). Alternative splicing is regulated by the synergy between trans-acting RBPs and cis-regulatory elements (22). RBM10 is a kind of alternative splicing factor that is enriched in the splicing sites at the 5′ and 3′ ends of introns and exons of pre-mRNA, with more abundant binding near the 3′ splicing site than those of the 5′ splicing site. By binding to small nuclear ribonucleoprotein (snRNPs) and cleavage sites of pre-mRNA substrates, RBM10 can synergistically remove introns and increase exon jumping events by more than 74% (23, 24). The change in RBM10 abundance and activity can lead to changes in some gene splicing patterns and the occurrence of many diseases, such as LUAD (25).

In addition to alternative splicing, RBM10 participates in at least three processes related to RNA expression, including pre-mRNA splicing, mRNA stabilization, and mRNA transcription. For example, RBM10 combines with *angiotensin receptor type 1* (*AT-1*) transcripts to improve transcriptional stability and downregulate *AT-1* expression (26). As we know, RBP can regulate the intracellular localization of non-coding RNA, methylation modification, the formation of miRNA silencing complex, alternative splicing and so on. As a member of this family, although there is very little research on RBM10 and non-coding RNA, we speculate that RBM10 must have the function of regulating non-coding RNA and will become the focus of future research. Using ICLIP, 90% of RBM10 are bound to RNA encoding protein, and the rest are bound to non-coding RNA. RBM10 has also been shown to bind non-coding RNAs, such as spliceosomal small nuclear RNAs, U2, and U12 (23), which can provide new ideas for future research. RBM10 may also participate in epigenetic regulation of gene transcription through post-translational modification of histones. This finding indicates that RBM10 is not only a splicing molecule but also involved in many aspects of RNA transcription and expression. By calibrating the expression of RBM10, it is theoretically possible to alter the development of cancer, and thus offer new therapeutic alternatives. However, only a few studies focus on the pharmacological implications of RBM10 (27).

## Roles of RNA-Binding Motif Protein 10 in Regulating Tumor Development

**Figure 2:**
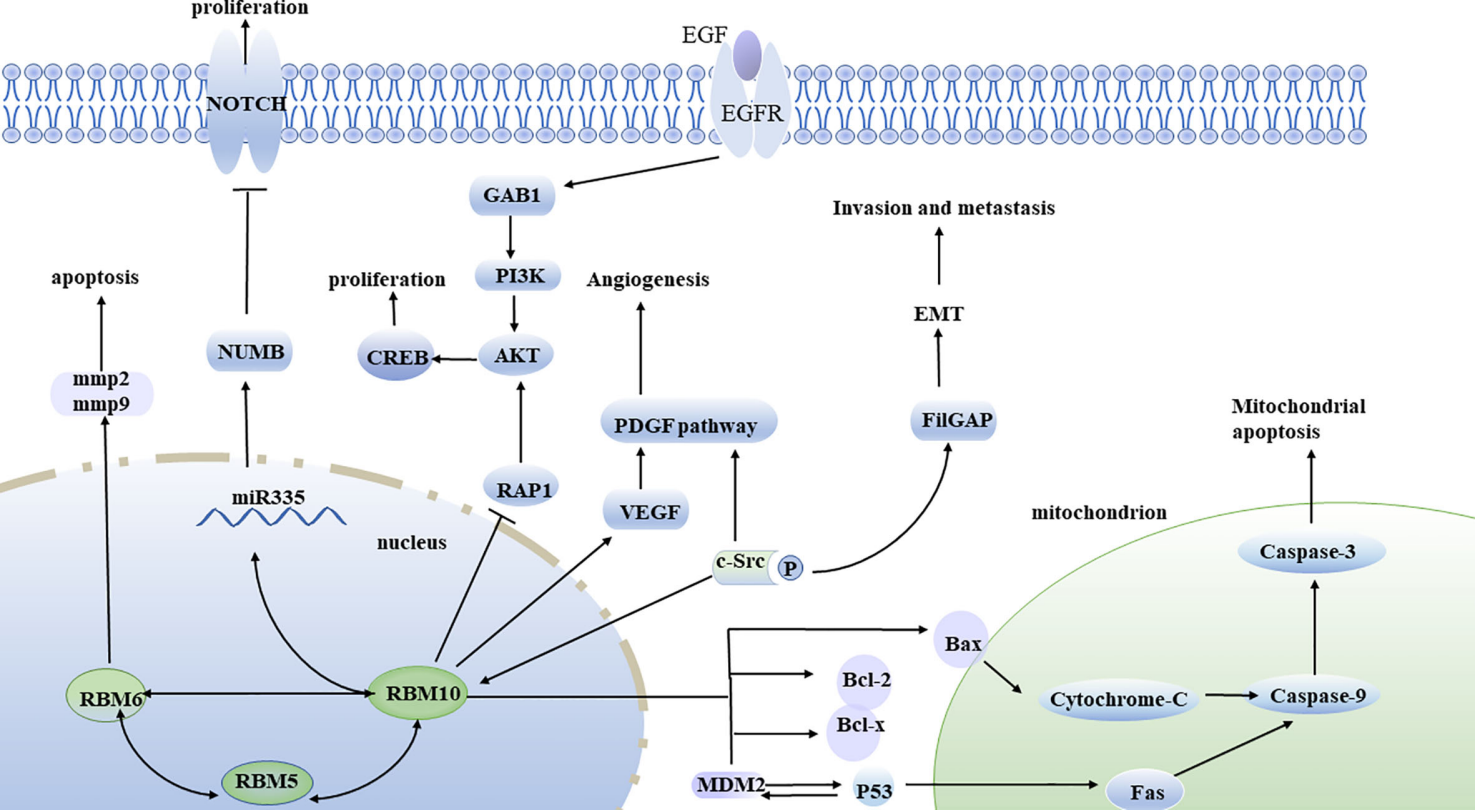
Molecular pathways of RBM10 discovered so far. *RBM10* overexpression inhibits cell proliferation through activation of the RAP1/AKT/CREB pathway. RBM10 binds to c-SRC tyrosine kinase, regulates FilGAP, promotes EMT, cell invasion, and metastasis. *RBM10* mutations are associated with the expression of VEGF, which then activates the PDGF pathway and angiogenesis. The c-SRC tyrosine kinase phosphorylates RBM10, which is then mobilized from the nucleus to the cytoplasm. Inhibition of RBM10 expression leads to downregulation of Numb, which can activate Notch pathway and promote tumor growth and proliferation. RBM10 also activates the apoptosis pathway by affecting the MDM2 and p53 negative feedback loop. RBM10 induces upregulation of Bax, promotes the release of cytochrome-c, activated caspase-3, and caspase-9 to activate the mitochondrial apoptosis pathway. RBM5, RBM6, and RBM10 can interact with each other.

RBM10 is widely recognized as a tumor suppressor gene because it can inhibit tumor proliferation, promote apoptosis, and prevent metastasis (**Figure 2** **)**. However, recent studies have also reported that RBM10 also plays a role in promoting tumor growth through an unknown mechanism.

### Apoptosis

RBM10 can regulate the selective cleavage of *Fas* transcripts (28). Fas is a classical death receptor involved in the Fas/Fas ligand (FasL) apoptosis pathway, which has anti-tumor activity (29), and can cause cascade reactions that lead to cell death. Fas is a pro-apoptotic protein that produces pro-apoptotic subtypes and anti-apoptotic subtypes through alternative splicing (30). Although RBM10 can cause the jumping of exon 6 in *Fas* that regulates its alternative splicing (31), this regulation has no cell specificity. BCL-X is an apoptosis-related protein that can be selectively cut into a shorter pro-apoptotic subtype, BCL-X (s), and a longer anti-apoptotic subtype, BCL-X (L). RBM10 can promote the selection of 5′-splice sites in exon 2 of the BCL-X precursor mRNA (32). A study of RBM10 in 122 breast cancer samples showed that the expression of RBM10 gene was positively correlated with the expression of the pro-apoptotic gene *Bax (* 33) and pro-apoptotic factors caspase-3 and caspase-9 (34), but negatively correlated with the expression of gene Bcl-2. As the final executor of apoptosis, caspase-3 can cause a cascade reaction in the cytoplasm by cleaving its downstream targets that trigger apoptosis. In particular, RBM10 promotes apoptosis by regulating the alternative splicing of apoptotic proteins. RBM10 can inhibit cell proliferation and induce apoptosis by activating the p53 pathway. P53 is an important tumor suppressor, and mutations in the *P53* gene have been found in more than 50% of human tumor tissues, making it the most common gene mutation found in malignant tumors. Gene defects in the p53 family may lead to spontaneous tumor development, embryo death or serious tissue abnormalities, which indicates that the activity of p53 family must be strictly regulated in order to maintain normal cell function (35). MDM2 forms the basis of p53 activation and creates a negative feedback loop of MDM2-p53. RBM10 can prolong the half-life of p53 by blocking the MDM2-p53 feedback loop and inhibiting the ubiquitination of p53, which has an anti-tumor effect (36). However, this action can only affect the protein level of p53 and does not affect mRNA levels. Thus, the regulation of p53 by RBM10 is at the post-translational level.

### Proliferation

RBM10 inhibits the Notch signaling pathway by alternative splicing of Numb gene and affects cell proliferation (16). Numb is a tumor suppressor, and the Notch pathway is the key proliferation pathway affected in breast cancer (37). The Notch signaling pathway affects many processes of normal cell morphogenesis, including differentiation, apoptosis in pluripotent stem cells, cell proliferation, and cell boundary formation (38). Besides, the low expression of Numb in breast cancer is associated with poor prognosis (39). The RRM2 of RBM10 recognizes C-rich sequences that can interact with intron 3 and exon 9 of Numb and promote the jump of exon 9 (15, 16). When RBM10 expression is downregulated, Numb is also downregulated, which can lead to the activation of the Notch pathway, enhanced HeLa cell lines colony formation, and promotion of tumor growth and proliferation (40). Additionally, miR-335 overexpression regulates the expression of Numb-L in endometrial carcinoma by targeting RBM10, providing a new biomarker for the diagnosis of endometrial cancer (41).

RBM10 inhibits cell proliferation through the RAP1/Akt/CREB signaling pathway (42). RBM10 inhibits the expression of CREB through a specific EPAC/RAP1A signal. RAP1A is an important regulator and mediator of Ras function, and its activation is related to a variety of cancers (43, 44). RBM10 can also reduce the activation of RAP1A and decrease phospho‐AKT and phospho‐CREB to inhibit tumor proliferation. This pathway is independent of MAPK/ERK and p38/MAPK, and CREB is highly expressed in many tumors. By evaluating the expression of key markers in these pathways that are closely related to the expression of GTP binding RAP1. By evaluating the expression of key markers in these pathways that are closely related to the expression of GTP binding RAP1, there was no significant change in the expression of phosphorylated ERK1/2 (Thr202/Tyr204) and phosphorylated p38 MAPK (Thr180/Tyr182) in RBM10 over expression and knockout cells. These results suggest that RBM10 mediated cell proliferation is not dependent on MAPK/ERK and p38 MAPK signaling pathways.

### Invasion and Metastasis

In the EGF stimulated cells, the EGFR is activated; Src family kinases are also activated (45). The Src family of kinases is involved in regulating cell proliferation, survival, adhesion, and migration (46). C-Src is a member of this family that can directly phosphorylate tyrosine residues in RBM10, which induces RBM10 to move from the nucleus to the extracellular matrix (47). It has been shown that Src family tyrosine kinase signaling regulates FilGAP through its association with RBM10 (48), and that this process may be downstream of the EFGR pathway. The positive correlation between the mRNA expression of *RBM10* and vascular endothelial growth factor (VEGF; an important angiogenesis promoter) supports the role of RBM10 in the regulation of angiogenesis (33). In addition, in a study of 19 cases of bronchoalveolar carcinoma (BCA), tissues ranging from *in situ* carcinoma to invasive carcinoma exhibited mutations in *RBM10* (49). Therefore, we speculate that RBM10 is also involved in inhibiting the invasion and metastasis of cancer cells (48). Gene set association analysis using the sequence permutation (GSAASeqSP) program revealed that the most enriched gene set following RBM10 knockdown (RBM10KD)in GLC20 cells was “hypoxia,” while the other three enrichment affected by hypoxia were “mTORC1 signal,” “epithelial mesenchymal transition” (EMT) and “angiogenesis” (50). However, it is unclear whether RBM10KD specifically promotes or inhibits these processes.

## Regulation of RNA-Binding Motif Protein 10 Expression

RBM10 has two different functions. It may act as a tumor suppressor, but also play a cancer-promoting role. To determine the reasons for these differential functions, we must understand what factors regulate the expression of RBM10 and what changes the final expression product of RBM10. To this end, two aspects should be considered: 1) RBM10 self-selective splicing and self-regulation and 2) RBM10 regulation by its homolog, RBM5.

### Self-Regulation of RNA-Binding Motif Protein 10

RBM10 can bind its pre-mRNA, thereby affecting gene expression and promotion of NMD. The 5′ splice sites of introns 6 and 12 of the RBM10 transcript are combined, resulting in the deletion of exon 6 or exon 12. The lack of mutations in exon 6 or exon 12 is the targets of NMD, indicating that overexpression of RBM10 will eventually negatively affect RBM10 mRNA and protein levels, which forms a negative feedback expression mechanism for RBM10 itself (51). Using RNA immunoprecipitation and next-generation sequencing technology, the RBM10 protein was shown to specifically bind to the RNA of the *RBM10v2* variant, indicating that RBM10 can bind its prerequisite RNA to regulate its expression (50). This self-regulation is possible because RBM10 is an RBP and, therefore, can affect its alternative splicing.

### RNA-Binding Motif Protein 10 Is Regulated by Its Homolog RNA-Binding Motif Protein 5

Previous studies demonstrated that both RBM10 and RBM5 enhance exon skipping in target genes, suggesting that RBM5 and RBM10 have compensatory regulation. RBM5 and RBM10v1 protein expression was significantly elevated in primary breast tissue and also showed a significant positive correlation (52). Furthermore, decreased RBM5 protein levels correlated with increased RBM10 in sham versus injured mouse brain homogenates (53). In addition, RBM10 can also down-regulate the mRNA and protein expression of RBM5 and its own, through alternate splicing-coupled nonsense-mediated mRNA decay (AS-NMD). For example, RBM10 can promote the jumping of exons 6 and 12 in *RBM10* transcripts and the jumping of exons 6 and 16 in *RBM5* transcripts. RBM10 overexpression down-regulates the expression of *RBM5* mRNA, while in the RBM10 knockdown (KD), *RBM5* expression is upregulated (51). So, RBM10 and RBM5 may perform reciprocal regulation.

## RNA-Binding Motif Protein 10 and RNA-Binding Motif Protein 10 Tumor Inhibitory Effects

RBM5, RBM6, and RBM10 have homologous structures at the protein level; therefore, their functions are hypothesized to be similar to one other. Understanding the function of RBM5 and RBM6 will help us gain a deeper understanding of the RBM10 and RBP families.

RBM5 is also known as G15, H37, and luca-15. It is a constituent gene of the 3p21.3 tumor suppressor region in humans. The protein localizes in the nucleus and can bind to DNA and RNA (54). RBM5 participates in the selective splicing of pre-mRNA by regulating the expression of some apoptosis-related proteins, including caspase 2, TNF-α, cell surface death receptor Fas/CD95, and p53. Thus, RBM5 can activate the mitochondrial apoptosis pathway and regulate cell apoptosis (55, 56). Previous studies have shown that downregulation of RBM5 can activate the Wnt/*β*-catenin signaling pathway and participate in lung epithelial injury induced by cigarette smoke extract (57). RBM5 is also involved in the downregulation of EGFR, which leads to the suppression of lung tumor cell proliferation, angiogenesis, invasion, and metastasis (58). Conversely, low expression of RBM5 can lead to the primary tumor and lymph node metastasis. Furthermore, the abnormal expression of RBM5 protein is associated with the occurrence of breast cancer (52), vestibular schwannoma (59), and renal cell carcinoma (60). RBM5 changes normal bronchial cells, promotes the development of tumor and changes the curative effect in patients. Compared with tumor cell lines, the expression level of RBM5 is higher in normal tissues, indicating that RBM5 has an anti-tumor effect. The decrease of RBM5 expression can increase the invasiveness of lung cancer, that is, the increase of nodule number and tumor size (58). Above all, RBM5 inhibits proliferation, affects cell cycle progression, and promotes apoptosis, which are generally considered components of the tumor-suppressing process.

The RNA-binding motif protein 6 (RBM6) is also known as 3g2, G16, def3, def-3, hlc-11, and nylu-12 and is located in the 3p21.3 tumor suppressor region in humans. Changes in *RBM6* mRNA expression can significantly increase the incidence rate of breast cancer (61), malignant tumors, ovarian cystadenoma, non-Hodgkin lymphoma, and pancreatic insulinoma (62). The expression of *RBM6* is downregulated in laryngeal cancer tissues and cell lines, while its overexpression promotes cell apoptosis, changes in *MMP-2* and *MMP-9* expression levels, and the upregulation of *caspase-3* expression. EGFR is a receptor that promotes tumor occurrence and development; its expression in tumor tissues is higher than that in adjacent and normal tissues. High expression of *RBM6* decreased the expression of the protein levels of EGFR, ERK, and p-ERK *in vivo* and *in vitro* (63). All these results indicate that RBM6 is a tumor suppressor; however, research on RBM6 is still very limited, and more is needed in the future to fully characterize RBM6 function in disease systems.

## RNA-Binding Motif Protein 10 and its Role in Human Disease

### RNA-Binding Motif Protein 10 and Human Cancers

#### Osteosarcoma

Osteosarcoma is a common malignant bone tumor in children and adolescents. Patients who recur similarly fare poorly with 10-year overall survival of 20% or less. The prognosis of patients with osteosarcoma has not changed for decades (64). The expression of RBM10 protein can reduce the proliferation of primary chondrocytes by inducing apoptosis in osteosarcoma cells through inhibiting the expression of Bcl-2, and promoting the expression of caspase-3 as well as the production of TNF-α (4). RBM10 overexpression can reduce the proliferation and colony formation of osteosarcoma cells and inhibit migration and invasion. After the outstanding achievements made in the 1970s and 1980s, despite the two cycles of surgical treatment, immunotherapy, and the current therapeutic approach, which involves neoadjuvant chemotherapy with two cycles of cisplatin (MAP), the prognosis of patients was still not significantly improved. Therefore, as a new possible targeted drug, RBM10 may be helpful to improve the prognosis.

#### Breast Cancer

Breast cancer is the most common cancer in women and the second most fatal tumor-based human disease (65). The expression of RBM genes (RBMX, RBM3, and RBM10) on the X chromosome, as well as the expression of apoptosis-related genes, namely Bcl-2 and Bax, was detected by differential RT-PCR in 122 cases of breast cancer. RBM10 highly correlates with the expression of Bax, the core gene in the caspase apoptosis pathway in breast cancer (33). Moreover, RBM10 was also positively correlated with the expression of caspase 3 and p53. The pcDNA-RBM10 vector was transfected into human non-small cell LC cell line A549. Then the expression of p53, caspase3, and caspase9 was detected by western blot (35). These results indicate that RBM10 plays a vital role in controlling apoptosis in breast cancer cells. In addition, the expression of two RBM10 variants was significantly correlated with the expression of VEGF (33). Therefore, RBM10 can mediate apoptosis in breast cancer cells, suggesting that it may be a useful target for breast cancer therapies.

#### Lung Cancer

For the past 30 years, lung cancer is the leading cause of cancer-related deaths worldwide (66). Mutations in *RBM10* that decreased its expression are most commonly associated with LUAD (34), while the overexpression of *RBM10* can inhibit lung cancer cell proliferation (36). Additionally, the RBM10 gene knockdown promotes cell proliferation *in vitro*, while the accumulation and stable overexpression of RBM10 in BALB/C nude mice significantly inhibited tumor growth. Thus, mutations in RBM10 are associated with tumor stage, lymph node metastasis, and poor survival rate, and therefore, there is potential in exploring the use of RBM10 as a biomarker for the progression and prognosis of LUAD. Most of the experiments in lung cancer considered RBM10 could promote apoptosis and inhibit proliferation. However, RBM10 has also been reported to promote tumors in a few studies. Compared with normal lung cells and tissues, the expression of RBM10 in LUAD cells and LUAD tissues was upregulated (2). The overexpression of RBM10 decreased the expression of the pro-apoptotic proteins Bax and caspase-8 but increased the expression of the anti-apoptotic protein Bcl-2. RBM10 overexpression also reduced the expression of p53, inhibiting the apoptosis pathway. The downregulation of *RBM10* expression resulted in decreased cell proliferation and growth, as well as decreased invasion and metastasis (2). We speculate the opposing conclusion may be related to different experimental conditions, or attributed to the effect of RBM10 variants.

Some studies suggest that the expression of *RBM10* is positively correlated with the expression of *EGFR* and that the MAPK and PI3K signaling pathways activated by EGFR are positively correlated with *RBM10* expression. It is hypothesized that the differential expression of *RBM10* mutations could affect disease prognosis. However, in the majority of the reports, RBM10 is still considered to play an anti-cancer role, with only a few suggesting it promotes the development of lung cancer. Therefore, we still maintain that RBM10 is a tumor suppressor that plays a role in lung cancer. In the future, we need to study what causes these two opposing effects of RBM10 on cancer regulation and development.

#### Liver Cancer

Hepatocellular carcinoma (HCC) is one of the highest incidence rate and mortality rates (67). Compared with normal tissues, EGFR protein expression is upregulated in HCC tissues; however, in HCC cells overexpressing *RBM10*, EGFR is downregulated. Overexpression of *RBM10* can reduce the protein levels of EGFR and p-ERK, inhibit the proliferation of HCCs, and induce apoptosis, while also inhibiting cell invasion (5). As a tumor suppressor gene, *RBM10* can reduce the malignant progression of HCC and is a novel prognostic biomarker and therapeutic target in patients with liver cancer.

#### Pancreatic Cancer

In the analysis of 109 cases of pancreatic cancer where *RBM10* mutations were present, tumors were all of a high grade, pT3 stage, and harbored lymph node metastasis in three of four cases. Furthermore, whole-exome sequencing of pancreatic cancer indicated that in 75% of these cases, *RBM10* mutations were associated with favorable patient prognoses, suggesting these mutations could improve the survival rate and prolong the survival period (6). However, a previous study found that the *RBM10* mutations could lead to a poor prognosis of metastatic diseases and a high risk of cancer recurrence. Mutations in *RBM10* are found in a third of cancers, including colon and pancreatic cancer (68).

#### Female Genital Cancer

RBM10 also has different roles in the development of female genital tumors. Gene analysis revealed that stable *RBM10* knockdown in HeLa cells was associated with a significant increase in colony formation. Furthermore, *RBM10* deletion inhibited apoptosis through the selective splicing of Fas and promoted the occurrence and development of tumors. The miR-335 expression is increased in endometrial tumor tissues and negatively correlated with RBM10 protein levels. However, there was no significant change in the level of *RBM10* mRNA, suggesting that the expression of *RBM10* may be regulated by post-transcriptional regulatory factors such as miRNAs (41). Angiogenesis plays an essential role in the pathogenesis of gynecological diseases such as endometriosis and malignant tumor. Folkman’s basic theory points out that every tumor more than 1–2 mm^3^ must enter the vascular system to grow further. Neutrophils can participate in angiogenesis by releasing a large number of VEGF stored in the cells (69). *RBM10* is also downregulated in endometrial carcinomas and can regulate the selective cleavage of VEGFA, thus affecting angiogenesis (70).

#### Colorectal Cancer

In addition to the tumors studied above, RBM10 is commonly mutated in colorectal cancer (71) and also plays an important role in colon cancer. Moreover, overexpression of *RBM10* is associated with increased disease invasiveness in patients with metastatic melanoma (72); however, studies in these tumors are still limited, and additional research is warranted. We have summarized the progress of RBM10 research in available tumor experiments in **Table 1**.

**Table 1:** Research progress of RBM10 in various tumors.

cancer	Tumor tissue number	Cell	RBM10 expression or mutation in cancer tissue	RBM10’s function	Affect pathway or key molecules	Reference
Breast carcinoma	73	MDA-MB231	High expression	Poor prognosis (+)	HER-2 (+)	(52)
	108		RBM10 mutation	Apoptosis	Caspase3 BAX	(73)
				Angiogenesis	VEGF CD105	
		MDA-MB231	RBM10 overexpression Or knockdown	metastasis	Src family tyrosine kinase FiLGAP	(48)
LUAD		A549	RBM10 mutation	Proliferation (+) (Antagonism of RBM5 and RBM6)	NOTCH pathway	(40)
	25	A549	Low expression	Apoptosis (+)	Bcl-2 (−) Caspase9, PARP (+)	
	90	A549 H1299	High expression Oncogene	proliferation, Invasion and metastasis (+) apoptosis (−)	P53(−) EGFR, MAPK PI3K/AKT pathway	(2)
	50		Exon10 mutation	Tumor stage, lymph node metastasis, survival rate		(25)
		A549 H1299 Xenograft	Overexpression of RBM10	Proliferation (+)	RAP1/AKT/CREB	(42)
		H460	Overexpression of RBM10	Apoptosis (+) Proliferation (−)	MEM2-P53 feedback	(36)
Osteosarcoma		U2OS	Overexpression of RBM10	Apoptosis (+) Proliferation And metastasis (−)	Bcl-2, TNF-α Caspase-3	(4)
BCA	19		RBM10 mutation	metastasis		(49)
CRC	619		mutation			(71)
PDA	109		RBM10 mutation	Prognosis survival rate (+)	KRAS	(6)
Endometrial cancer	29	HEC-1-A RL95-2	Low expression	Angiogenesis	VEGFA	(70)
	47		Target of miR335		miR335/Numb-L	(41)
Hepatocellular carcinoma		HepG2	Assembling PLK4-STIL complexes	Proliferation Tumorigenesis	Centromere loss regulates chromosome division	(74)
		HepG2	Low expression	Prognosis (+) Apoptosis (+) Proliferation and metastasis (-)	Caspase3(+) EGFR, p-ERK (−)	(5)
CCA	66		Mutation			(75)

LUAD, Lung Adenocarcinoma; BCA, bronchioloalveolar carcinoma; PDA, Pancreatic ductal adenocarcinoma; CRC, Colorectal carcinoma; CCA, Cholangiocarcinoma; (+), positive correlation (-); negative correlation.

### RNA-Binding Motif Protein 10 and Non-Tumor Diseases

RBM10 mutations can not only affect the progress of cancer but also cause other, non-oncogenic diseases. When mutations in *RBM10* become pathogenic, they can affect the long-term survival rate of patients with talipes equinovarus, which is an atrial septal defect (TARP) syndrome (76) that is an X-linked recessive disease characterized by the Robin sequence, congenital heart disease, growth retardation, eating difficulties, and horseshoe varus (77). A large number of parallel sequencing of exons on the X chromosome confirmed that RBM10 is a gene associated with cleft palate syndrome. Moreover, RBM10 also regulates the AT1 receptor gene expression through transcriptional and post-transcriptional mechanisms (7), which affects the pathogenesis of atherosclerosis (26). In heart disease, the deletion of *RBM10* can cause H9c2 cell hypertrophy (78), while the splicing-independent function of RBM10 controls specific 3′UTR processing that regulates cardiac hypertrophy (79) and can be reversed by ectopic inhibition of RBM10. It was found that RBM10 controlled the proper splicing of DNA (cytosine-5)-methyltransferase 3B (DNMT3b) and increased the expression level of the enzymatically active DNMT3b2, but decreased the expression of the *DNMT3b3* splicing isomer (80). Both DNMT3b isomers can effectively bind to NF-*κ*B and regulate the development of inflammation, and these results suggest that RBM10-mediated DNMT3b2 regulation plays an important role in NF-*κ*B-mediated transcription induction. Therefore, RBM10-dependent DNMT3b regulation may be a therapeutic target for many inflammatory diseases (80).

Dengue virus targets RBM10 and reduces splicing and innate immune response to host cells (81). SAT1 is a spermidine/spermine acetyltransferase, which can reduce the storage of polyamines in cells and limit virus replication. After infection, the RBM10 protein level was low, which led to SAT1 exon 4 skipping. Dengue polymerase NS5 interacts with RBM10 and its expression alone can trigger RBM10 proteasome-mediated degradation. The overexpression of RBM10 in infected cells prevented the splicing change of SAT1 and restricted virus replication. The downregulation enhanced the splicing switch and facilitated virus replication, which revealed the antiviral effect of RBM10. These studies indicate that RBM10 plays an important role in cancer and non-cancer diseases.

### Possible Mechanism for the Opposing Effects of RNA-Binding Motif Protein 10 on Tumors

In this review, we have described the two main variants of RBM10, RBM10v1, and RBM10v2, which have 49 and 54% identity and can bind different RNAs (52). Based on the review of the literature, we speculate that these two variants are likely to affect the differential function observed in the study of RBM10. RBM10v2 has a common target greater homology with RBM5 than RBM10v1 and RBM5 as an example of the dichotomous functions of these variants, the RBM5-RBM10v2 complex regulates cell cycle progression and promotes apoptosis, while RBM10v1 inhibits tumor apoptosis in breast cancer. RBM10v1 expression in breast cancer specimens correlated with the expression of proapoptotic BAX and the tumor suppressor gene p53 (50). Furthermore, we hypothesize that the proportional differences between these two RBM10 variants can have two different effects on tumor promotion and inhibition. However, it is still unclear why these two variants are present in different proportions in tumor cells, and more in-depth research is required to understand what upstream regulatory mechanisms underpin *RBM10* expression.

The decrease of RBM5 expression may be a key step in the development of small cell lung cancer, because RBM5 regulates many transformation related processes in small cell lung cancer cells. RBM5 is similar to RNA binding protein RBM10 in structure and function. These two proteins have tumor inhibitory effects in many tumor-cell lines. This review also highlights that RBM10 has six times more targets than RBM5 (50). Moreover, RBM5 and RBM10 have 401 common targets, indicating that RBM5 and RBM10 work together during the progression of tumor development. Therefore, the effect of RBM10 on tumors is likely to be related to RBM5. Due to their similarity and recent evidence that RBM10 mutates in up to 21% of lung cancer, majority of studies hypothesized that RBM10 has the tumor suppressive properties of RBM5 in small cell lung cancer. However, through transcriptome analysis and functional analysis, also found that the function of RBM10 was contrary to the hypothesis; in GLC20 SCLC cell lines with endogenous RBM5 deletion, RBM10 actually promoted cell proliferation and other transformation related processes. Using RNA immunoprecipitation next generation sequencing (RIP-seq) and western blotting, studies have demonstrated that RBM5 regulates RBM10 expression at post-transcriptional level through direct interaction with specific RBM10 splicing variants (50). Indeed, as shown with experiments using an RBM5-NULL cell line that does not express RBM5, RBM10 upregulated EMT and angiogenesis when compared to their downregulation in the presence of RBM5. These results illustrated that RBM10 function could not only be altered but also actually reversed when coupled with RBM5 to exert tumor-inhibiting functions.

Although most RBM10 mutations resulted in a decrease in its expression, in specific tumor types or developmental stages, these mutations also may lead to an increase in RBM10 expression and promote tumor growth. This may also be related to the phosphorylation of RBM5 and RBM10. Nevertheless, it is suggested that non-phosphorylated RBM protein may be pro-apoptotic (2). In tumor tissues, the upregulation of RBM10 and RBM5 may occur due to increased translation and protein stability, resulting in a significant increase in protein content, especially if RNA content remains unchanged or decreased. The c-Src family tyrosine kinases can phosphorylate RBM10, and the phosphorylated RBM molecules are reportedly upregulated in tumor tissues, which may affect the localization and function of RBM10. Similarly, the upregulation of Src family tyrosine kinases can also promote the transfer of RBM10 from the nucleus to cytoplasm (2).

## Clinical Significance of RNA-Binding Motif Protein 10

The second major reason of death around the globe is cancer. Cancer was responsible for approximately 8.8 million deaths (1). For some patients who lost the chance of surgery in late stage, targeted drugs are almost the only treatment. At present, the targeted drugs in cancer treatment on the market is still very limited, most of which are KRAS target (30%), followed by EGFR (15%), and a few others such as ALK (5%), HER2 (2%) and so on. Besides, many patients develop resistance to conventional radiotherapy, chemotherapy, and targeted drug resistance. Therefore, it is imperative to find new biomarkers for early diagnosis, prognosis evaluation, and create new targeted drugs. The studies reviewed here have shown that deletion or mutation of *RBM10* is associated with the occurrence and poor prognosis of human cancers, including lung and pancreatic cancer (6, 34). For example, *RBM10* mutation is significantly associated with the American Joint Committee on Cancer (AJCC) stage diagnostics, lymph node metastasis, and male patients, but not with smoking, age, tumor size, or differentiation (25). Mutations in exon 10 of *RBM10* can significantly promote the proliferation and invasion of tumor cells, and the 5-year survival rate of patients with these mutations was significantly lower than that observed in healthy individuals (36.4 *vs*. 46.5% of RBM 10 wild type; χ2 = 5.466, P = 0.019) (25). However, some *RBM10* mutations are also related to the improvement in survival rate (6), suggesting that our understanding of the specific pathogenic patterns of RBM10 mutagenesis is still limited.

**Figure 3:**
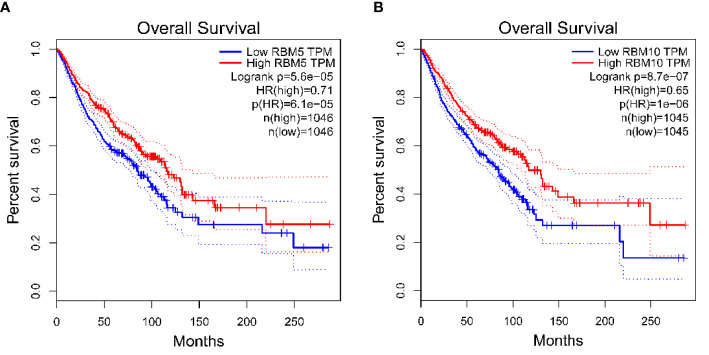
RBM 5 and RBM10 function influences patient survival rates in different diseases. **(A)** Effect of RBM5 on the survival rate of four kinds of carcinoma, including BRCA, Breast invasive carcinoma; LUAD, Lung adenocarcinoma; LIHC, Liver hepatocellular carcinoma; and PAAD, Pancreatic adenocarcinoma. **(B)** Effect of RBM10 on the survival rate of four kinds of carcinoma, including BRCA, LUAD, LIHC, and PAAD. The results showed that the survival rates in patients with upregulated (the red line) RBM5 **(A)** and RBM10 **(B)** were both significantly higher than those in patients with downregulated (the blue line) RBM5 **(A)** and RBM10 **(B)**. The red lines and the blue lines represent the survival rates when the gene is upregulated and down-regulated respectively. The dotted lines represent the 95% confidence interval.

We analyzed the effect of RBM10 and RBM5 protein expression on prognosis and survival rate in breast cancer, LUAD, pancreatic cancer, and hepatocellular carcinoma by GEPIA (http://gepia.cancer-pku.cn/). The results showed that the survival rate in patients with upregulated *RBM*5(**Figure 3A**) and *RBM10* (**Figure 3B**) was significantly higher than that in patients with low expression of these genes. In conclusion, *RBM10* mutations can be used as an independent prognostic factor for a variety of prominent cancers affecting patient survival and quality of life. However, there is still limited clinical data on the diagnostic ability of RBM10, which shall be tackled in future research.

## Prospective Future of RNA-Binding Motif Protein 10 Research

Loss of function of *RBM10* mutations is common in many cancer patients. Although we know that RBM10 may act as a tumor suppressor, it not only promotes apoptosis and inhibits proliferation but is also related to cell invasion and metastasis because it can reduce glycolysis, EMT, and angiogenesis (16, 34, 48). However, the specific mechanism and the upstream and downstream regulatory factors of RBM10 function are still unknown. In particular, previous studies (28, 42) focused on apoptosis and proliferation rather than on the specific role of RBM10, which is still uncharacterized. In addition, the specific mutations of *RBM10* observed in different tumors, how they lead to changes in the expression of other genes and proteins, why some mutations lead to either tumor proliferation or inhibition, and whether these differences are related to the two RBM10 variants need further examination. In addition, we now posit that as an RBP, the effect of RBM10 on non-coding RNA should be considered. At present, most of the published studies focus on the role of RBM10 in the pathogenesis and molecular mechanism of diseases, but few studies have been applied to clinical practice. Therefore, our future goal is to verify the role of RBM10 in disease diagnosis, treatment, prognosis, and risk assessment. We need to identify the molecules upstream and downstream of RBM10 and its pathway, and design new targeted drugs to provide new therapeutic options for patients with targeted drug resistance.

## Author Contributions

KW contributed to the conception and design of the study. YC and QZ reviewed and analyzed the literature. YC produced the main draft of the manuscript. YC and XD made figures. KW and RL obtained funding for the study. All authors contributed to the article and approved the submitted version.

## Funding

This study was supported by the Natural Science Foundation of Jilin Province (20180101103JC to RL), the Technology Research Funds of Jilin Province (20190303162SF to KW), and the Medical and Health Project Funds of Jilin Province (20191102012YY to RL).

## Conflict of Interest

The authors declare that the research was conducted in the absence of any commercial or financial relationships that could be construed as a potential conflict of interest.
